# Psychiatrists’ attitudes towards functional neurological disorders: results from a national survey

**DOI:** 10.3389/fpsyt.2023.1216756

**Published:** 2023-07-14

**Authors:** Angela Marotta, Antonio Lasalvia, Mirta Fiorio, Enrico Zanalda, Guido Di Sciascio, Claudia Palumbo, Davide Papola, Corrado Barbui, Michele Tinazzi

**Affiliations:** ^1^Department of Neurosciences, Biomedicine and Movement Sciences, University of Verona, Verona, Italy; ^2^Department of Mental Health ASL TO3 and AOU San Luigi Gonzaga, Collegno, Italy; ^3^Department of Mental Health, ASL Bari, Bari, Italy

**Keywords:** functional neurological disorders, conversion disorders, psychosomatic medicine, psychiatric practice, education, survey

## Abstract

**Introduction:**

Functional neurological disorder (FND) presents motor, sensory, and cognitive symptoms characterized by clinical signs incongruent with known neurological disease. Together with other health professionals, like neurologists, psychiatrists can play an essential role in diagnosing and managing these disorders. Hence, understanding their opinion and clinical experience with FND is of utmost importance to catch potential educational needs and improve healthcare services for patients. This study aims at assessing the knowledge, opinion, and clinical approach of Italian psychiatrists to FND.

**Methods:**

Members of the Italian Society of Psychiatry completed a 14-item web-based survey investigating their approach to FND. Results. Overall, 174 questionnaires were completed. Our main findings suggest that Italian psychiatrists have a psychogenetic conceptualization of FND. “Conversion disorders”, in fact, is the term most frequently used by Italian psychiatrists to refer to FND, thus implying a psychological etiology of these disorders. Congruently with this view, psychotherapy associated with pharmacological therapy is considered the most appropriate treatment by psychiatrists, while physiotherapy is an under-recognized treatment option for FND.

**Discussion:**

The present study highlights that a psychogenetic view of FND dominates among Italian psychiatrists. This could be due to out-of-date knowledge about the pathophysiology of this group of disorders. Promoting education about novel approaches to FND would be of crucial importance to improving care for patients suffering from this condition.

## 1. Introduction

Functional neurological disorders (FND) present with motor, sensory, or cognitive symptoms which are inconsistent over time and are characterized by clinical signs that are incongruent with known neurological disease (1, 2). Since the beginning of the 19th century, these disorders have been conceived as primarily psychiatric illnesses arising from the *conversion* of psychological distress into physical symptoms (3). Congruently, the diagnosis was based on the identification of psychological causes, and psychiatrists were considered the leading health professionals for this group of disorders.

Research on the pathophysiology of FND has recently challenged such a perspective, moving FND away from a psychogenic conceptualization toward a biopsychosocial model (4, 5). The model acknowledges the complexity of FND by identifying neurobiological (e.g., intellectual disability, acute physical pain, physiological arousal), psychological (e.g., personality disorders, panic attacks, hypervigilance), and social variables (e.g., chronic illness in the family, loss of employment, provider diagnostic uncertainty) that can increase the vulnerability to develop FND (predisposing factors), cause the onset of symptoms (precipitating factors) and maintain (perpetuating factors) the disorder once it has been established (4, 5). In particular, recent studies shed new light on the neurobiological and cognitive underpinnings of FND, suggesting that functional neurological symptoms could be explained by dysfunction across different brain networks, which in turn affect specific domains, like attention (6), executive functioning (7, 8), sense of agency (i.e., feeling of control over voluntary movements) (9–11), and emotion processing (11, 12). The diagnosis of FND shifted from exclusion to a rule-in approach including positive signs of inconsistency (e.g., symptoms vary in frequency and intensity over time) and incongruency with other neurological conditions (13). Further, the requirement for preceding psychological stress and for exclusion of feigning has been discarded from diagnostic criteria.

The novel diagnostic approach to FND better fits with the expertise of neurologists, which are more trained than psychiatrists in physical examination and classification of neurological disease (14). Nonetheless, a diagnosis based only on the identification of positive physical signs leaves apart an in-depth assessment of illness beliefs, personality traits, and psychosocial factors that are critically involved in the pathophysiology of FND (14, 15). Psychiatrists are well-equipped to evaluate these factors, thus substantially improving case formulation. Moreover, psychiatrists’ expertise in assessing predisposing, precipitating, and maintaining factors is essential to develop tailored therapeutic plans for patients (14, 16). Last but not least, the biopsychosocial model is foundational to psychiatry and, as mentioned above, is now the prevailing model through which FND is explained.

Despite their potential role in improving the diagnosis and treatment of FND, little is known about the opinions and clinical experiences of psychiatrists regarding these disorders. A recent study by Dent et al. (17) found that the conversion model of FND still predominates among psychiatrists, thus suggesting that a novel conceptualization of FND as due to a multifaceted etiology has not been entirely embedded in their approach to these disorders. It would therefore be interesting to evaluate how this view translates into attitudes and clinical practice of psychiatrists with patients suffering from FND. The present study aims to address this issue by surveying psychiatrists’ knowledge, opinion, and clinical approach to FND. The current study is part of a larger research project involving Italian health professionals (18, 19) that treat patients with FND in their clinical practice. Diagnosis and management of FND require an interdisciplinary approach in which a comprehensive assessment of psychiatric, neurological, cognitive, and psychosocial factors guides the development of patient-centered treatment plans able to address the complexity of FND (5, 20). This approach calls for a multidisciplinary team involving neurologists, psychiatrists, and other health professionals (e.g., general practitioners, psychologists, and physiotherapists) (5, 20). Understanding the attitudes and clinical experiences of each of these professional figures in treating patients suffering from FND is of utmost importance to promote a common language and strengthen cooperation among health professionals in the management of FND, thus optimizing healthcare service and delivery.

## 2. Materials and methods

### 2.1. Survey

The survey was based on the methodology used in our previous study conducted on a sample of Italian neurologists (19). The questionnaire consisted of fourteen single and multiple-choice questions included in two main sections. One section regarded demographics (age, sex, geographical area of residence) and professional characteristics (years of post-specialization and practice setting). The second section assessed psychiatrists’ knowledge, opinions, and clinical practice when treating patients with FND, with a specific focus on terminology, explanation of symptoms, predictors of diagnosis, treatment, and the role of psychiatrists in the diagnosis and treatment of FND. All the survey questions are reported in the Supplementary materials.

The survey was conducted among the members of the Italian Society of Psychiatry (SIP). The invitation to participate was sent by e-mail to all potential respondents (*N* = 690) by the General Secretary of the SIP using the 2020 members list. The e-mail explained that the survey was meant to investigate opinions, knowledge, and clinical experience with non-organic neurological disorders among psychiatrists. As in our previous studies (18, 19), we chose the term non-organic to avoid connotations with “functional” or “psychological” mechanisms underlying the disease. To exclude potential bias due to misleading terminology, we provided an example of what we meant by a non-organic disorder (e.g., neurological symptoms, like tremor, which may disappear with diverted attention). A survey link was embedded in the e-mail and allowed direct access to the questionnaire. Respondents start completing the questionnaire after giving their consent.

The Google Forms Online tool (Google LLC, Menlo Park, CA, United States) was used to collect responses over a period of 8 weeks (1 February – 29 March 2022). Two e-mail reminders were sent to the SIP members, 2 and 6 weeks after the initial mailing. The study received ethical approval from the University of Verona (CARP) and was conducted in accordance with the Declaration of Helsinki.

### 2.2. Data analysis

Survey responses were examined with descriptive statistics, including frequencies and percentages. Chi-squared test was used to analyze gender distribution. Statistical significance was set at *p* < 0.05. All the analyzes were performed using SPSS software (version 19).

## 3. Results

### 3.1. Demographical data and professional characteristics

Overall, 174 out of 690 contacted psychiatrists (response rate, 25%; mean age ± standard deviation (SD), 48 ± 14; mean years of practice ± SD, 18 ± 14), with a balanced number of males (55%) and females (45%) (chi-squared = 1.47, *p* = 0.22) completed the questionnaire (Table 1). The majority were specialist psychiatrists (*n* = 145, 83%) while the remaining were residents (*n* = 29, 17%). Most were employed in the public sector, either in a community mental health center (*n* = 76, 44%) or in an inpatient psychiatric unit (*n* = 42, 24%).

**Table 1:** Sample demographics and years of practice.

	Responses – no. (%)
Sex	
Male	95 (55)
Female	79 (45)
Age (years)	
< 40	59 (34)
41–50	37 (21)
51–60	30 (17)
>60	48 (28)
Years of practice	
<10	67 (38)
11–20	33 (19)
21–30	33 (19)
>30	41 (24)

### 3.2. Opinions, knowledge, and clinical experiences with FND

#### 3.2.1. Practice with FND patients

Half of the sample (*n* = 89,51%) stated that less than 10% of the patients seen in a week presented with FND. Thirty-eight percent (*n* = 66) reported a higher proportion of FND (10–25%) and a few (*n* = 10, 6%) stated that 25–50% of their patients have a diagnosis of FND. Very few (*n* = 2, 1%) stated that more than half of their patients have FND or were unable to estimate how many of their patients might have a FND (*n* = 7, 4%; Table 2).

**Table 2:** Exposure to patients with FND and terms chosen to define the condition.

	Responses – no. (%)
Percentage of patients with FND seen in 1 week	
<10	89 (51)
10–25	66 (38)
25–50	10 (6)
>50	2 (1)
Do not know	7 (4)
Terminology*	
Conversion disorder	87 (50)
Somatoform disorders	83 (48)
Functional neurological disorders	82 (47)
Psychogenic disorder	42 (24)
Non-organic disorder	23 (13)
Unspecific anxiety syndrome	21 (12)
Hysteria	15 (9)
Stress-related syndrome	11 (6)
Depression	7 (4)
Medically unexplained disorder	5 (3)

FND, functional neurological disorders; *More than one response allowed.

#### 3.2.2. Terminology

Respondents could choose from a list of 10 terms they usually used to describe functional neurological symptoms (Table 2). Sixty-one respondents (35%) selected more than one term. “Conversion disorder” (*n* = 87, 50%) was the most frequently used term to describe FND, followed by “Somatization disorders” (*n* = 83, 48%), “Functional Neurological Disorders” (*n* = 82, 47%), and “Psychogenic disorder” (*n* = 42, 24%). Less frequently used terms were: “Non-organic disorder” (*n* = 23, 13%), “Unspecific anxious syndrome” (*n* = 21, 12%), “Hysteria” (*n* = 15, 9%), “Stress-related disorder” (*n* = 11, 6%), “Depression” (*n* = 7, 4%), and “Medically unexplained disorder” (*n* = 5, 3%). Very few chose the “I do not know” answer (*n* = 5, 3%).

#### 3.2.3. Probability that patients simulate symptoms

Most respondents believed that simulation (e.g., intentional production of symptoms) was little (*n* = 123, 71%) or moderately probable (*n* = 27, 16%) in this kind of disorder, while 12% (*n* = 21) found it not at all probable, and very few stated that simulation was highly probable (*n* = 3, 2%).

#### 3.2.4. Explanation of symptoms

When asked about their preferred way to explain symptoms to patients with FND, the majority chose “psychogenic disorder” (*n* = 69, 40%). Many chose “disorder due to abnormal functioning of the nervous system” (*n* = 61, 35%), while few chose “absent neurological disorder” (*n* = 23, 13%) or “stress” (*n* = 14, 8%). Other explanations were: “somatization,” “physical symptoms of emotional distress,” and “poor diagnostic investigation.”

#### 3.2.5. Predictors of diagnosis

**Figure 1:**
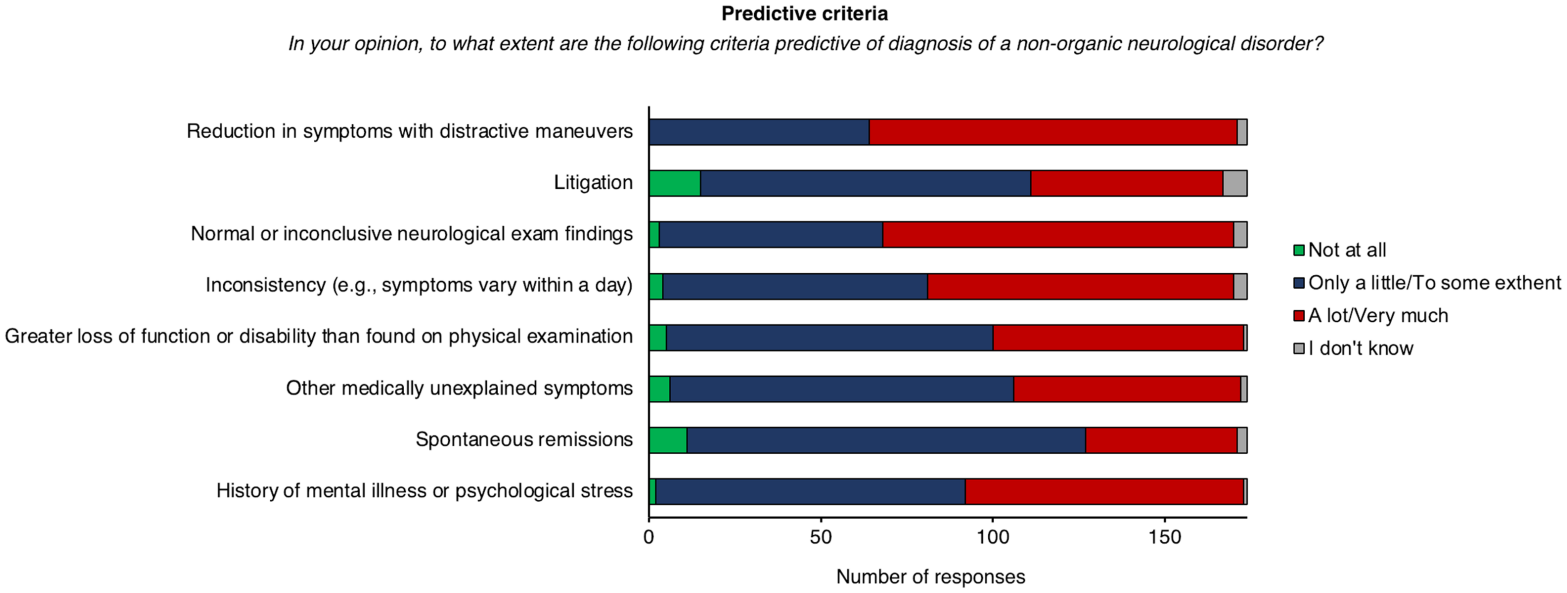
Distribution of responses for predictors of diagnosis of FND.

When asked to judge the extent to which certain diagnostic criteria were predictive for FND (from “not at all” to “very much”), most respondents (*n* = 107, 61%) reported that “reduction in symptoms with distractive maneuvers” represents a predictive feature of this group of disorders, followed by “normal or inconclusive neurological examination findings” (*n* = 102, 59%) and “inconsistency” of symptoms (e.g., symptoms vary over time) (*n* = 90, 52%; Figure 1). Conversely, “spontaneous remissions” (*n* = 116, 67%), “other medically unexplained symptoms” (*n* = 100, 57%), “litigation” (*n* = 96, 55%), “greater loss of function or disability than found on physical examination” (*n* = 95, 55%), “previous mental illness or psychological stress” (*n* = 90, 52%) were considered as poorly predictive (Figure 1).

#### 3.2.6. Specialist consultation and treatment

**Figure 2:**
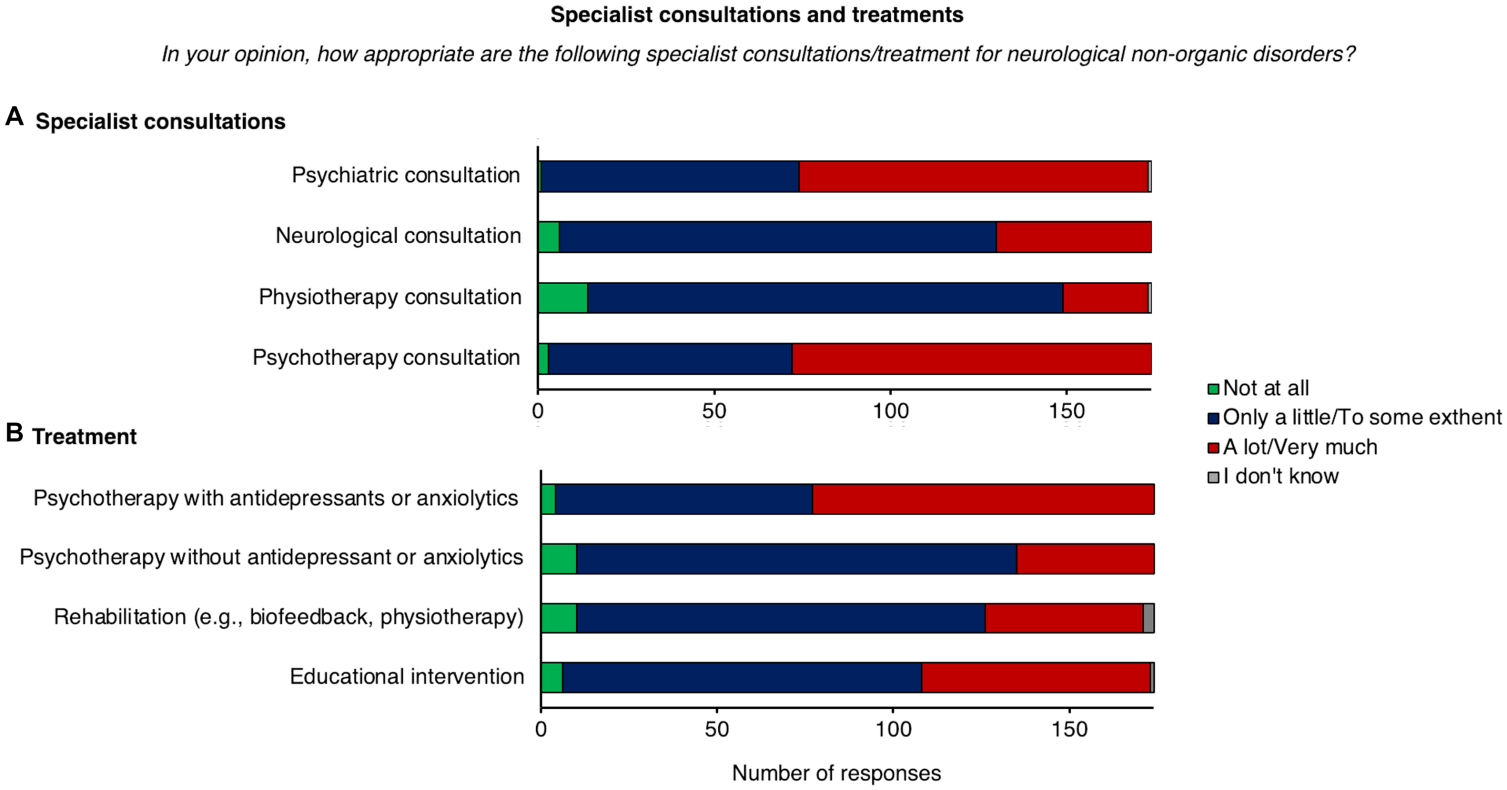
Distribution of responses for specialist consultation and treatment. **(A)** Specialist consultations. **(B)** Treatment.

When psychiatrists were asked to rate the degree of adequacy of specialist consultations for FND (from “not at all” to “very much”), “psychotherapy consultation” (*n* = 102, 59%), and “psychiatric consultation” (*n* = 99, 57%) were frequently rated as “a lot” or “very much” adequate for FND. On the other hand, “physiotherapist consultation” (*n* = 135, 78%) and “neurological consultation” (*n* = 124, 71%) were prevalently rated as “only a little” or “to some extent” adequate for FND patients (Figure 2A).

When psychiatrists were asked to indicate the degree of adequacy of five different treatments for FND, “psychotherapy with antidepressant or anxiolytic medications” (*n* = 97, 56%) was most frequently rated as “a lot” or “very much” appropriate for FND patients (Figure 2B). Conversely, “pharmacological treatment” (*n* = 140, 80%), “psychotherapy without antidepressant or anxiolytic medications” (*n* = 125, 72%), “rehabilitation (e.g., biofeedback, physiotherapy)” (*n* = 116, 67%), and “educational interventions” (*n* = 102, 59%) were frequently rated as “only a little” or “to some extent” adequate.

#### 3.2.7. Management strategies

**Figure 3:**
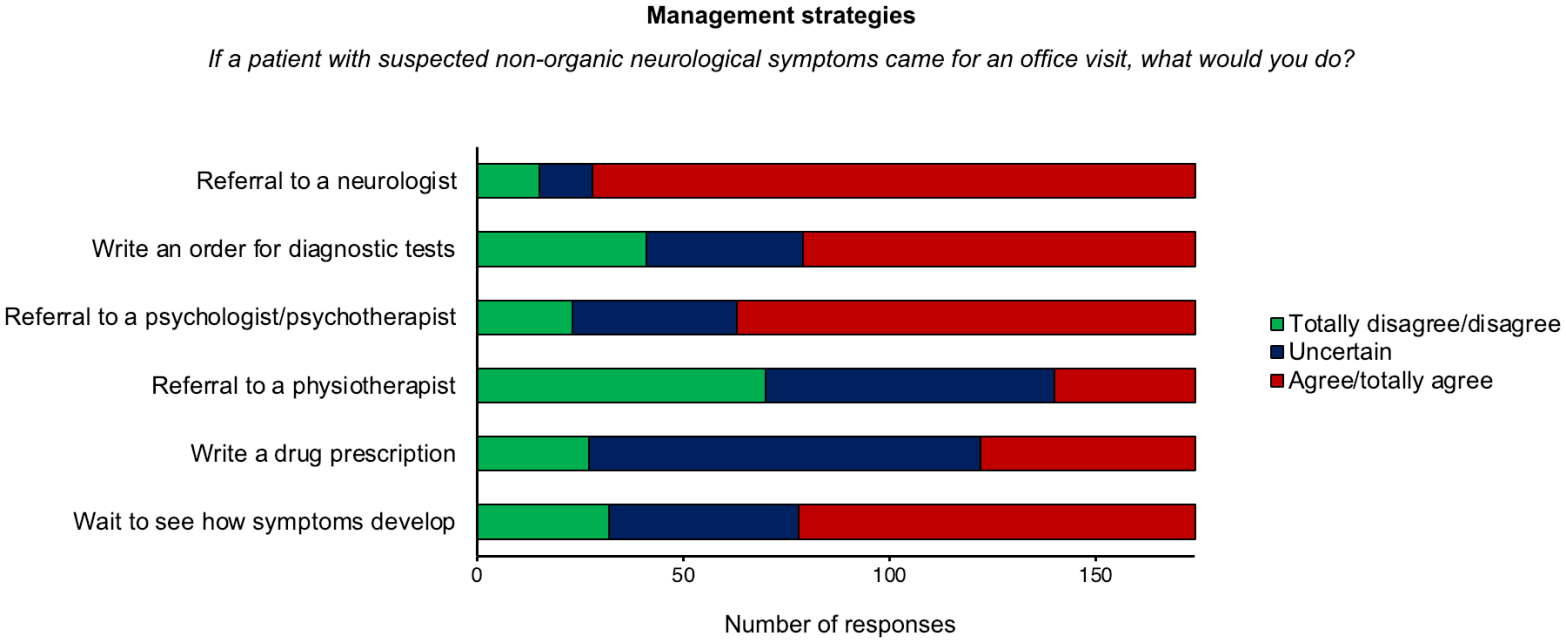
Distribution of responses for management strategies.

When asked to indicate their level of agreement (from “totally disagree” to “totally agree”) with management strategies, most respondents “agreed” or “totally agreed” that patients with FND should be referred to a “neurologist” (*n* = 146, 84%), and to a “psychologist or psychotherapist” (*n* = 111, 64%); more than half of the sample reported that the most appropriate strategy would be “wait and see how symptoms develop” (*n* = 96, 55%), and to prescribe “instrumental examinations” such as magnetic resonance imaging (*n* = 95, 55%). The management strategy most frequently excluded from clinical practice was “referral to a physiotherapist” (“totally disagree” or “disagree”: *n* = 70, 40%). Half of the sample was undecided about “Pharmacological prescription” (*n* = 95, 55%; Figure 3).

#### 3.2.8. Satisfaction

When asked to rate their satisfaction in managing FND on an 11-point scale from 0 (not at all) to 10 (extremely satisfied), most respondents (*n* = 134, 77%) rated their level of satisfaction between 5 and 10, with an overall average score of 5.91 (SD,2.15).

#### 3.2.9. Role of psychiatrist

When asked about the clinical role of psychiatrists in the management of patients with FND, most of respondents (*n* = 129, 74%) gave more than one response, with “following-up the treatment together with other specialists (neurologist, psychotherapist, physiotherapist)” as the most frequent (*n* = 153, 88%), followed by “educational intervention for patients and their families” (*n* = 90, 52%), and “make a diagnosis and recommend adequate treatment (e.g., physiotherapy, psychotherapy, pharmacological treatment)” (*n* = 75, 43%). Nineteen percent (*n* = 33) of the sample reported that psychiatrists should “make a diagnosis and personally follow-up the patient,” while very few (*n* = 16, 9%) thought that the role of psychiatrist should be simply to refer FND patients to a specialist for their medical condition.

## 4. Discussion

This survey investigated the attitudes and clinical experiences of Italian psychiatrists with patients with FND. Our main findings unveil a still prevalent psychogenic conceptualization of FND embedded in the diagnostic and clinical approach to the patients.

The term “functional neurological disorder” was among the most frequently chosen term in our sample. Of note, previous studies suggested that this term is preferred by patients since it reduces the fear of social stigma, which is historically related to a psychiatric conceptualization of FND (21–24). Nonetheless, an even higher proportion of respondents used the term “Conversion disorder” to name FND. This term evokes the Freudian conversion model of FND, by which symptoms are conceived as a physical sign of emotional distress (25). Congruently, many respondents in our sample (40%) prefer to explain FND in terms of psychogenic disease, conveying a psychological etiology of symptoms. Importantly, many patients felt unbelieved when the explanation of symptoms is based on psychological factors. Patients’ medical history does not always reveal traumatic experiences or psychological difficulties in FND and, even when present, it is difficult to understand the mechanisms by which past experiences can have determined actual symptoms (3). Moreover, recent findings on neurobiological and cognitive correlates of FND, allowed to identify other potential risks and precipitating factors, like attentional dysregulation (6), and deficits in motor planning, intention, execution or inhibition (10, 26–30). This evidence allowed a transition from the conversion model of FND in which psychological factors are central in the etiology of the disease to a multidimensional model, involving multiple triggering and predisposing risk factors (15, 31). As part of this transition, psychological factors were removed as diagnostic criteria from the DSM-5, and a multidisciplinary approach to diagnosis and treatment is now highly recommended (13). Many psychiatrists in our sample were inclined to explain FND symptoms using a psychiatric and psychological terminology. This approach might be due to the fact that psychiatrists usually evaluate patients after the neurological examination. At this stage, the diagnostic process still lacks an exploration of psychosocial factors that can be provided by the psychiatrist through an in-depth investigation of psychiatric comorbidities, personality traits, illness belief, and other psychological and social variables which act as risk and maintaining factors of the disorder. Nonetheless, together with previous studies, our findings suggest that many psychiatrists are anchored to the conversion model of FND, as revealed by the frequent use of psychological-related terms, and of a psychogenic conceptualization of the disorder (17, 32). Such a model has dominated psychiatry for a long time and shaped the education of health professionals on FND in the last decades. This, together with a lack of up-to-date training on the diagnosis and management of FND, could explain the actual approach of psychiatrists to these disabling conditions. Expanding on these findings, our study highlights that a psychological conceptualization of FND determines a clinical approach that is mainly oriented toward psychotherapy and psychiatric intervention. More precisely, respondents in our sample thought that psychotherapists and psychiatrists are the most adequate health professionals for FND. Congruently, psychotherapy associated with pharmacological treatment is believed to be the most appropriate treatment for these patients. This approach partly overlaps with the novel conceptualization of FND which embraces emotional and psychological dimensions as potential risks and maintaining factors of FND (15). Thus, a proper understanding of the patient’s condition would require an in-depth assessment of psychological factors and, when needed, an hoc-psychotherapy and psychiatric intervention (14). Recent studies demonstrated that psychotherapy is a valuable approach for FND, being effective in reducing symptoms’ severity and improving psychological well-being in different subtypes of FND patients (33–37). However, a large body of evidence suggests that other therapeutic options (e.g., diagnostic explanation, physiotherapy, occupational therapy, and multidisciplinary rehabilitation) could also be useful for managing FND symptoms (38–42). Among these, physiotherapy is considered a valuable treatment for functional movement disorder (FMD), one of the most common subtypes of FND (13). Physiotherapy for FMD is usually provided after an in-depth neurological and psychiatric assessment (43), and includes education on the disease, a demonstration that normal movement can occur, retraining with diverted attention, and challenging maladaptive behaviors (e.g., use of adaptive equipment, like crutches) (40, 41). A growing body of evidence proved efficacy of this approach for improving physical function in FMD (40, 41, 44, 45). Despite this, as in our previous studies involving general practitioners and neurologists (18, 19), physiotherapy was rated as the least appropriate for FND, thus implying that it is still poorly recognized as a valuable approach to FND. These findings highlight the need to promote knowledge of different therapeutic options for FND among health professionals, thus enhancing patient care.

Another important observation is that although some psychiatrists believe it unlikely that patients deliberately produce their symptoms, a high percentage (89%) of respondents hypothesize that symptoms might be feigned (i.e., patients simulate their symptoms) with a little, moderate, or high probability. Similar results emerged also from our previous study involving neurologists (19) and might indicate out-of-date knowledge of the pathophysiology of the disease among health professionals. Accumulating evidence suggests that FND symptoms (especially motor symptoms) can be due to an altered sense of agency, that is the feeling of controlling voluntary movements (11, 28, 46–48). For instance, neuroimaging studies found reduced activation of brain regions involved in the sense of agency, which may be associated with a lack of subjective feeling of control over voluntary movements in patients with FND [e.g., (9, 11, 28)]. Promoting knowledge of these pathophysiological mechanisms would improve psychiatrists’ understanding of FND, thus in turn resulting in a more proficient way to deal with these disorders. Suspicion about feigning could also be due to a lack of diagnostic instruments to clearly distinguish FND from malingering. Thus, further research is needed to develop *ad hoc* tests to exclude deception in FND.

In line with the current knowledge of the disease, psychiatrists believe that changes in symptoms with distractive maneuvers and inconsistency over time are the most predictive factors for a diagnosis of FND. These findings suggest that psychiatrists can recognize the typical signs of FND symptoms. However, as a first step in the management of FND, most respondents would ask for a neurological consultation. This approach might be driven by the need for excluding other neurological conditions by means of *ad hoc* neurological examination. This is in line with current guidelines, suggesting that neurological assessment is needed for establishing a diagnosis of FND (13). Neurologists are well-trained in the evaluation of physical signs and can distinguish FND symptoms from other neurological diseases. On the other hand, psychiatrists are well-trained in the clinical assessment of psychosocial factors which contribute to symptom development and maintenance (15, 46). Thus, patients would benefit from closer collaboration between these two health professionals, especially in the diagnostic phase.

However, comparing the current survey with the previous one conducted on neurologists (19) it seems that we are still far from an integrated approach to FND involving a collaboration between psychiatrists and neurologists. For instance, from a qualitative analysis of the results of the two surveys, we found that these two health professionals consider each other to be, respectively, not adequate for FND. Indeed, many psychiatrists believe neurologists are not appropriate for managing FND, and, vice versa, many neurologists thought that psychiatry is rarely useful for FND. In line with a previous study (17), these findings are suggestive of a distance between psychiatrists and neurologists, which can be due to different conceptualizations of the disease. Indeed, while psychiatrists hold primarily on psychological models to explain FND (17), neurologists seem to prefer a neurobiological explanation of the disease, which mostly excludes psychological factors (17). In both cases, these health professionals do not endorse a comprehensive view of FND integrating neurobiological, psychological, and social factors. Nonetheless, both psychiatrists and neurologists are open to a multidisciplinary approach to the disorder as evidenced by the fact that both psychiatrists and neurologists thought their role is to follow up the treatment with other specialists. Educational interventions could enhance collaboration between different health professionals, thus improving a multidisciplinary approach to FND. For instance, the interaction between specialties could be optimized by training psychiatrists to perform a clinical assessment of physical symptoms for FND, while neurologists should be trained to better recognizing psychiatric comorbidities (2, 49, 50). Also, promoting the adoption of shared terminology and explanation of symptoms is needed to enhance consistency across health professionals in the communication of diagnosis to patients.

Some limitations need to be acknowledged. It is possible that only psychiatrists interested in FND responded to the survey, thus reducing the generalizability of our findings. Furthermore, the limited number of questions might have precluded a more comprehensive understanding of the respondents’ attitudes and knowledge about FND. These limitations notwithstanding, this survey provided novel insights on the knowledge, opinion, and clinical experience of a sample of Italian psychiatrists with FND. Together with our previous studies involving general practitioners and neurologists (18, 19), the results of this survey suggest that up-to-date knowledge on FND is still lacking among health professionals in our country. Specific educational courses are needed to improve knowledge about these disorders and promote a multidisciplinary approach to FND, thus in turn improving both primary and specialist care for patients (51).

## Data availability statement

The raw data supporting the conclusions of this article will be made available by the authors, without undue reservation.

## Ethics statement

The study involving human participants was reviewed and approved by CARP - University of Verona. The participants provided their written informed consent to participate in this study.

## Author contributions

MT, AM, and MF: conceptualization and methodology. AM, AL, EZ, GDS, CP, and CB: recruitment of participants and data collection. AM: data curation and visualization. AM, MT, MF, AL, CB, and DP: analysis and interpretation of data. AM: writing – original manuscript. AM, MT, MF, AL, CB, DP, GDS, CP, and CB: writing – review and editing. All authors contributed to the article and approved the submitted version.

## Conflict of interest

The authors declare that the research was conducted in the absence of any commercial or financial relationships that could be construed as a potential conflict of interest.

## Publisher’s note

All claims expressed in this article are solely those of the authors and do not necessarily represent those of their affiliated organizations, or those of the publisher, the editors and the reviewers. Any product that may be evaluated in this article, or claim that may be made by its manufacturer, is not guaranteed or endorsed by the publisher.
