# Supplementary material for: Psychiatrists’ attitudes towards functional neurological disorders: results from a national survey
Source: Front Psychiatry. 2023 Jul 14;14:1216756. doi: 10.3389/fpsyt.2023.1216756 (PMC10375048; doi:10.3389/fpsyt.2023.1216756)
Supplement: Supplementary file 1 [file Data_Sheet_1.DOCX]

Supplementary Material

Psychiatrists’ attitudes towards functional neurological disorders: results from a national survey

**Angela Marotta, Antonio Lasalvia, Mirta Fiorio, Enrico Zanalda, Guido Di Sciascio, Claudia Palumbo, Davide Papola, Corrado Barbui, Michele Tinazzi**

*** Correspondence:**Dr. Angela Marotta
[angela.marotta@univr.it](mailto:angela.marotta@univr.it)

# Supplementary information: Survey Questions

**1. Age _______**

**2. Sex**

☐ Male

☐ Female

**3. Year of practice (post-specialization) _______**

**4. Place of residence _______**

**5. Practice setting**

☐ Community mental health center

☐ Outpatient clinic

☐ Inpatient psychiatric unit

☐ Other (specify)

**6. In your practice, how many patients do you see in a week who might present neurological symptoms without an organic cause?**

☐ <10

☐ 10 - 25

☐ 25 - 50

☐ >50

☐ Don’t know

**7. Which of the following terms do you usually use to describe a clinical case characterized by neurological symptoms without an organic cause? [You can indicate more than one answer]**

☐ Functional neurological disorders

☐ Somatoform disorders

☐ Non-organic disorder

☐ Psychogenic disorder

☐ Conversion disorder

☐ Stress-related syndrome

☐ Unspecific anxiety syndrome

☐ Depression

☐ Medically Unexplained Disorder

☐ Hysteria

☐ I don’t know

☐ Other (specify)

**8. In your opinion, how probable is it that a patient with a non-organic neurological disorder simulates his/her symptoms?**

☐ Not at all

☐ Low probability

☐ Moderate probability

☐ High probability

☐ Very high probability

**9. Which of the following explanations would you use to describe non-organic neurological symptoms to your patient?**

☐ Disorder due to abnormal functioning of the nervous system

☐ Absent neurological disorder

☐ Psychogenic disorder

☐ Stress

☐ Other (specify)

**10. In your opinion, to what extent are the following criteria predictive of diagnosis of a non-organic neurological disorder?**

|  | Not at all | Only a little | To some extent | A lot | Very much | I don’t know |
| --- | --- | --- | --- | --- | --- | --- |
| Reduction in symptoms with distractive maneuvers | ☐ | ☐ | ☐ | ☐ | ☐ | ☐ |
| Litigation |  |  |  |  |  |  |
| Normal or inconclusive neurological exam findings | ☐ | ☐ | ☐ | ☐ | ☐ | ☐ |
| Inconsistency (e.g., symptoms vary within a day) | ☐ | ☐ | ☐ | ☐ | ☐ | ☐ |
| Greater loss of function or disability than found on physical examination | ☐ | ☐ | ☐ | ☐ | ☐ | ☐ |
| Other medically unexplained symptoms | ☐ | ☐ | ☐ | ☐ | ☐ | ☐ |
| Spontaneous remissions | ☐ | ☐ | ☐ | ☐ | ☐ | ☐ |
| History of mental illness or psychological stress | ☐ | ☐ | ☐ | ☐ | ☐ | ☐ |

**11. In your opinion, how appropriate are the following specialist consultations/treatment for neurological non-organic disorders?**

|  | Not at all | Only a little | To some extent | A lot | Very much | I don’t know |
| --- | --- | --- | --- | --- | --- | --- |
| Psychiatric consultation | ☐ | ☐ | ☐ | ☐ | ☐ | ☐ |
| Neurological consultation | ☐ | ☐ | ☐ | ☐ | ☐ | ☐ |
| Physiotherapy consultation | ☐ | ☐ | ☐ | ☐ | ☐ | ☐ |
| Psychotherapy consultation | ☐ | ☐ | ☐ | ☐ | ☐ | ☐ |
| Pharmacological treatment of symptoms | ☐ | ☐ | ☐ | ☐ | ☐ | ☐ |
| Psychotherapy *with* antidepressants or anxiolytics | ☐ | ☐ | ☐ | ☐ | ☐ | ☐ |
| Psychotherapy *without* antidepressant or anxiolytics | ☐ | ☐ | ☐ | ☐ | ☐ | ☐ |
| Rehabilitation (e.g., biofeedback, physiotherapy) | ☐ | ☐ | ☐ | ☐ | ☐ | ☐ |
| Educational intervention | ☐ | ☐ | ☐ | ☐ | ☐ | ☐ |

**12. If a patient with suspected non-organic neurological symptoms came for an office visit, what would you do?**

|  | Totally disagree | Disagree | Uncertain | Agree | Totally agree |
| --- | --- | --- | --- | --- | --- |
| Referral to a neurologist | ☐ | ☐ | ☐ | ☐ | ☐ |
| Write an order for diagnostic tests | ☐ | ☐ | ☐ | ☐ | ☐ |
| Referral to a psychologist/psychotherapist | ☐ | ☐ | ☐ | ☐ | ☐ |
| Referral to a physiotherapist | ☐ | ☐ | ☐ | ☐ | ☐ |
| Write a drug prescription | ☐ | ☐ | ☐ | ☐ | ☐ |
| Wait to see how symptoms develop | ☐ | ☐ | ☐ | ☐ | ☐ |

**13. What is your level of satisfaction in managing a patient presenting with non-organic neurological symptoms?** [Select a number from 0 (no satisfaction) to 10 (high satisfaction)]

| No satisfaction |  | | | | | | | | | High satisfaction |
| --- | --- | --- | --- | --- | --- | --- | --- | --- | --- | --- |
| 0 | 1 | 2 | 3 | 4 | 5 | 6 | 7 | 8 | 9 | 10 |

**14. What is the psychiatrist’s role in the management of patients with non-organic neurological disorders? [You can indicate more than one answer]**

☐ Make a diagnosis and personally follow-up the patient

☐ Make a diagnosis and recommend appropriate treatment

☐ Refer the patient to a specialist for the condition

☐ Follow-up treatment together with other specialists (e.g., neurologist, physiotherapist, psychotherapist)

☐ Provide for education of the patient and family members

☐ Other (specify)
